# Development and validation of a machine learning model to predict hemostatic intervention in patients with acute upper gastrointestinal bleeding

**DOI:** 10.1186/s12911-025-02969-x

**Published:** 2025-03-24

**Authors:** Kajornvit Raghareutai, Watcharaporn Tanchotsrinon, Onuma Sattayalertyanyong, Uayporn Kaosombatwattana

**Affiliations:** 1 Division of Gastroenterology, Department of Medicine, Faculty of Medicine Siriraj Hospital, Mahidol University, Bangkok, 10700 Thailand; 2 Siriraj Informatics and Data Innovation Center, Siriraj Hospital, Mahidol University, Bangkok, Thailand; 3 Siriraj GI endoscopy Center, Siriraj Hospital, Bangkok, Thailand

**Keywords:** Machine learning, Upper GI bleeding, UGIB, Endoscopic intervention, Prediction

## Abstract

**Background:**

Acute upper gastrointestinal bleeding (UGIB) is common in clinical practice and has a wide range of severity. Along with medical therapy, endoscopic intervention is the mainstay treatment for hemostasis in high-risk rebleeding lesions. Predicting the need for endoscopic intervention would be beneficial in resource-limited areas for selective referral to an endoscopic center. The proposed risk stratification scores had limited accuracy. We developed a machine learning model to predict the need for endoscopic intervention in patients with acute UGIB.

**Methods:**

A prospectively collected database of UGIB patients from 2011 to 2020 was retrospectively reviewed. Patients older than 18 years diagnosed with UGIB who underwent endoscopy were included. Data comprised demographic characteristics, clinical presentation, and laboratory parameters. The cleaned data was used for model development and validation in Python. We conducted 80%–20% split sample training and test sets. The training set was used for supervised learning of 15 models using a stratified 5-fold cross-validation process. The model with the highest AUROC was then internally validated with the test set to evaluate performance.

**Results:**

Of 1389 patients, 615 (44.3%) of the cohorts received the endoscopic intervention (293 variceal- and 336 nonvariceal-bleeding interventions). Eighteen features, including demographic characteristics, clinical presentation, and laboratory parameters, were selected as input for 15 machine learning models. The result revealed that the linear discriminant analysis model could achieve the highest AUROC of 0.74 to predict endoscopic intervention. The model was validated with the test set, in which the AUROC was increased from 0.74 to 0.81. Finally, the model was deployed as a web application by Streamlit.

**Conclusions:**

Our machine learning model can identify patients with acute UGIB who need endoscopic intervention with good performance. This may help primary care physicians prioritize patients who need referrals and optimize resource allocation in resource-limited areas. Further development and identification of more specific features might improve prediction performance.

**Trial Registration:**

None (Retrospective cohort study)

**Patient & Public Involvement:**

None

**Supplementary information:**

The online version contains supplementary material available at 10.1186/s12911-025-02969-x.

## Background

Acute upper gastrointestinal bleeding (UGIB) remains a prevalent and significant medical condition encountered in routine clinical practice, with an annual incidence of 80–150 per 100,000 population and mortality rates spanning from 2% to 15% [1–3]. Given a considerable spectrum of severity, multiple international guidelines recommended pre-endoscopic risk stratification to assess and triage patients according to severity. Esophagogastroduodenoscopy (EGD) should be performed within a suggested time frame of 12–24 h from the onset of presentation [4–7]. Endoscopy is the most effective tool for diagnosing UGIB, and endoscopic therapy is indicated for lesions with high-risk stigmata to control bleeding and prevent rebleeding. For risk stratification, various scoring systems have been developed during the past two decades, such as the pre-endoscopic Rockall score (RS), Glasgow-Blatchford score (GBS), and AIMS65 score [8–10]. These scores aim to classify patients into low-risk or high-risk groups, thereby guiding the subsequent treatment strategy. Patients classified as a high-risk group carry higher mortality and rebleeding rates and, therefore, require in-hospital management. The pre-endoscopic Rockall score and the AIMS65 score were designed to predict mortality, while the GBS aimed to predict the likelihood of in-hospital management, including endoscopy, transfusion support, or surgery. Studies comparing the performance of these scoring systems revealed that the GBS has the highest performance and very high sensitivity [11–13]. However, the specificity was still limited [14, 15]. Furthermore, these scoring systems did not focus mainly on predicting hemostatic intervention.

The role of artificial intelligence (AI) in the medical profession is undergoing rapid expansion since machine learning can effectively handle large, complex, and heterogeneous datasets, extract correlations of parameters in detail, and accurately predict the outcome with experience [16–18]. As evidenced by numerous studies, the application of AI has yielded satisfying results in risk stratification, endoscopic findings, and mortality rate in UGIB [19–26]. In 2020, Seo et al. created a machine learning model to predict adverse events such as mortality, hypotension, and rebleeding in patients with initially stable nonvariceal bleeding. The model showed a higher ability to detect adverse events than conventional scores [27]. The other two models that focus on predicting blood transfusion or mortality for UGIB in the intensive care unit exhibited impressive performance, with an area under receiver operating characteristic (AUROC) exceeding 0.80 [22, 28]. Moreover, Shung et al. developed an available online machine-learning model from multicenter patient data to stratify low-risk patients who can be treated in an outpatient setting [24]. A multicenter study proved that the model performs better than the GBS, with great sensitivity and high specificity [29].

From our perspective, predicting the need for endoscopic intervention in patients with UGIB is one of the key decisions in management flow, and it would benefit physicians in the resource-limited area. This prospect could optimize the selective referral of patients from the primary healthcare center to the endoscopic center. Currently, only a few machine learning models precisely predict the need for endoscopic intervention in UGIB [30]. Then, we developed a simplified machine-learning model with structured data analysis for clinical decision-supporting systems to indicate the need for endoscopic intervention in patients with acute UGIB.

## Material and methods

### Patients

Prospectively collected data from adult patients who presented acute overt UGIB at Siriraj Hospital, Bangkok, Thailand, from January 2011 to December 2020 were retrospectively reviewed. The UGIB management protocol in our hospital followed international guidelines [4–7]. However, the final treatment decision was based on the attending physician and the bleeding team, including the endoscopist, radiological interventionist, and surgeon, who were available on a 24–7 basis. An expert endoscopist or trainees under close supervision performed the endoscopy. Endoscopic findings and hemostatic intervention were recorded using an Endosmart program. Hemostatic interventions for ulcers with high-risk bleeding [Forrest classification Ia (spurting bleeding), Ib (oozing bleeding), and IIa (non-bleeding visible vessel)] include a single or combination of adrenaline injection, hemostatic clip, thermal hemostasis, or hemostatic powder. For adherent clots, an attempt was made to remove the clot and examine the character of the underlying lesion. The intervention will not be applied in low-risk ulcers, including clean base ulcers and hematin spots. For variceal bleeding, rubber band ligation or glue injection was usually used for hemostasis of active bleeding lesions; for nonbleeding esophageal varices with high-risk stigmata, prophylaxis band ligation was performed depending on the endoscopist’s decision. The study included patients aged 18 years and older with acute UGIB who underwent EGD with comprehensive documentation of endoscopic findings. The inclusion was done by retrieving the patients from the Siriraj GI endoscopic center database using the search term “upper GI bleeding or UGIB” from the indication for EGD. The exclusion criteria were patients with in-hospital onset of UGIB, onset of UGIB more than 72 hours before hospital visit, and missing data on baseline characteristics, bleeding presentation, laboratory findings, endoscopic result, or intervention. Eligible patients who met the defined criteria were analyzed and used to develop and validate the machine-learning model.

### Data collection and outcome

The data from the chart and endoscopic record were extracted manually by well-trained GI fellows. They were divided into three categories: patient characteristic data, bleeding characteristic data, and laboratory data. All the data must be available before endoscopy. The baseline characteristics were extracted from the patient’s history, which was documented before the hospital visit. The bleeding presentation was noted by the first physician who encountered the patient, and laboratory tests were the initial test after the patient visited the hospital and before the GI consultation. Characteristic data of the patient included age, sex, co-morbidities documented in the medical file before the bleeding event, such as heart disease, stroke, chronic kidney disease, cirrhosis, and active malignancy, use of antithrombotic agents [conventional non-steroidal anti-inflammatory drugs (NSAIDs), cyclooxygenase-(COX) 2 inhibitors, aspirin, clopidogrel, and oral anticoagulants], previous episodes of UGIB and duration of bleeding before hospitalization documented by the first physician who encounters the patient. The definition of each comorbidity was described in Supplementary Material Appendix 1.

Characteristics of bleeding composed of the clinical manifestation of bleeding, such as type of vomitus (red or coffee-ground emesis) and type of stool (melena, maroon stool, red stool), the presence of syncope, altered consciousness (defined by a decreased Glasgow coma score less than 13 or documented as the chief complaint to the hospital), systolic blood pressure (SBP), heart rate, and need for resuscitation (fluid therapy with the rate of fluid higher than 500 ml/h without vasopressor or vasopressor needed after optimal fluid therapy).

Initial laboratory test data included hemoglobin, platelet count, blood urea nitrogen (BUN), creatinine, serum albumin, and international normalized ratio (INR). The BUN and creatinine were analyzed as BUN/creatinine ratio as it would diminish the falsely high BUN from renal causes such as pre-renal azotemia or chronic kidney disease. These parameters are used in previous scoring systems associated with hospital intervention and mortality [8–10]. The endpoint selected to develop the machine learning model was the requirement of endoscopic hemostatic intervention for variceal or non-variceal procedures, including epinephrine injection, hemostatic clip, thermal hemostasis, rubber band ligation, glue injection therapy, and hemostatic powder. All the data was cleaned and rechecked for exclusion criteria.

For the data pre-processing step, we adopted the One-Hot Encoding method for this experiment because most machine learning algorithms are not capable of handling categorical data without encoding. Furthermore, this experiment also adopted the normalization technique, which is the z-score, as part of the data pre-processing step for machine learning. This technique was used to rescale the values of numeric columns in the dataset without distorting differences in the ranges of values.

### Model development

For this study, we built and developed machine learning models in Python to predict the need for endoscopic intervention (Version 3.10, 64 bits). The data set of patients was randomly divided into two groups with an 80%–20% ratio as training and test sets. To reduce the risk of overfitting and ensure that the model can perform well in various samples, we applied a stratified 5-fold cross-validation as a validation technique. The training data were then fed to all models available in the model library using cross-validation to train and validate the models. These 15 supervised learning models included Linear Discriminant Analysis, Logistic Regression, Naïve Bayes, CatBoost Classifier, Extra Trees Classifier, Quadratic Discriminant Analysis, Random Forest Classifier, Gradient Boosting Classifier, Ada Boost Classifier, Light Gradient Boosting Machine, Extreme Gradient Boosting, K-Neighbors Classifier, Decision Tree Classifier, Dummy Classifier, and Lasso regression. They are available in a Python library called Pycaret (version 3.1.0). Since we adopted the stratified 5-fold cross-validation, the training data was randomly divided into five subsets or folds. The model was trained and evaluated five times, using a different fold as the validation set. Then, performance metrics from each fold were averaged to estimate the model’s performance, which was shown as the average area under the receiver operating characteristic (AUROC), accuracy, sensitivity, and specificity on the validation sets across five folds. Based on these comparison results, we determined the optimal model to predict the need for endoscopic intervention by selecting the model that could achieve the highest AUROC. Since default hyperparameters were implemented in all models, the best model was subsequently adjusted with a hyperparameter-tuning method in Pycaret to find the best prediction performance. In this experiment, we implemented a random grid search over a pre-defined grid search for hyperparameter tuning. In addition, we increased the number of iterations, ranging from 100 to 1000 at intervals of 100, to find the best performance. However, the same results were obtained, so the minimum number of iterations, which is 100, was chosen in this experiment. Then, the tuned model was internally validated with the test set to evaluate the performance and analyze the essential factors for the prediction. Finally, the model was deployed on the local host with the Python library, Streamlit. The outcome was shown as the need or the lack of a need for endoscopic intervention. The prediction probability was noted in the result as a percentage for the user to make decisions for further management.

### Statistical analysis

Qualitative data were analyzed by frequency and percentage, while quantitative data were analyzed by mean and standard deviation. The difference in variables between the two groups was analyzed using Fischer’s exact test or the Mann–Whitney U test. The prediction performance was measured by the AUROC curve analysis, sensitivity, specificity, and accuracy. The negative predictive value (NPV) and positive predictive value (PPV) of the model were calculated. The AUROC value was predefined as follows: acceptable threshold (≥ 0.7), fair performance (≥ 0.7 but < 0.8), good performance (≥ 0.8 but < 0.9), and excellent performance (≥ 0.9). A comparison of the AUROC of the proposed model and the conventional score was performed using a paired permutation test. A P-value < 0.05 was considered statistical significance. All statistical analyses were performed using Python (Version 3.10, 64 bits).

The sample size recruitment strategy was designed to include as many patients as possible to achieve a more efficient machine-learning model. This study followed the TRIPOD-AI reporting guideline and the ethical guidelines of the Declaration of Helsinki and was approved by the Siriraj Institutional Review Board (COA No. Si 1028/2021). The checklist of the TRIPOD-AI reporting guideline is provided in Supplementary Material Appendix 2. Since this was a retrospective analysis, informed consent was not obtained from the patients.

## Results

### Patient characteristic

The database of 2,201 patients with acute UGIB was reviewed. Among these, 635 patients with missing data, 170 patients with delayed hospitalization, and seven patients with in-hospital UGIB were excluded, resulting in 1,389 patients being eligible for model development. All patients underwent upper endoscopy within 120 h, and 615 (44.3%) of the cohorts received the endoscopic intervention; 293 variceal interventions, 336 nonvariceal interventions, and 14 patients received both variceal and nonvariceal interventions. The baseline characteristics, bleeding characteristics, and laboratory findings are presented in Table 1.

**Table 1:** Baseline characteristics, clinical presentation, and laboratory findings

	Intervention (n = 615)	No intervention (n = 774)	*P*-value
**Baseline characteristics**			
Age, mean (SD)	61.5 (14.6)	66.5 (15.1)	<0.001
Male, n (%)	430 (69.9)	472 (61.0)	<0.001
Heart disease, n (%)	80 (13.0)	199 (25.7)	<0.001
Stroke, n (%)	43 (7.0)	85 (11.0)	0.014
Cirrhosis, n (%)	315 (51.2)	122 (15.8)	<0.001
Chronic kidney disease, n (%)	85 (13.8)	155 (20.0)	0.003
Malignancy, n (%)	127 (20.7)	104(13.4)	<0.001
Previous UGIB, n (%)	131 (19.6)	140 (15.6)	0.047
NSAIDS, n (%)	121 (19.7)	175 (22.6)	0.207
COX-2 inhibitors, n (%)	4 (0.7)	3 (0.4)	0.706
Aspirin, n (%)	97 (15.8)	200 (25.8)	<0.001
Clopidogrel, n (%)	25 (4.1)	30 (3.9)	0.967
Oral anticoagulant, n (%)	49 (8.0)	129 (16.7)	<0.001
**Clinical presentation**			
Time to presentation (hours), mean (SD)	21.7 (21.1)	29.6(24.4)	<0.001
Red emesis, n (%)	269 (43.7)	141 (18.2)	<0.001
Coffee-ground emesis, n (%)	167 (27.2)	285 (36.8)	<0.001
Melena, n (%)	267 (43.4)	435 (56.2)	<0.001
Maroon stool, n (%)	52 (8.5)	52 (6.7)	0.263
Red stool, n (%)	13 (2.1)	11 (1.4)	0.437
Syncope, n (%)	165 (26.8)	187 (24.2)	0.283
Alter mental status, n (%)	21 (3.4)	24 (3.1)	0.861
Systolic BP, mmHg, mean (SD)	110.2 (23.3)	118.5 (25.9)	<0.001
Heart rate, bpm, mean (SD)	94.2 (19.5)	91.2 (19.1)	0.001
Resuscitation, n (%)			
•Intravenous fluid	170 (27.6)	120 (15.5)	<0.001
•Vasopressor	10 (1.6)	2 (0.3)	
**Laboratory findings**			
Hemoglobin, g/dL mean (SD)	8.1 (2.4)	8.2 (2.6)	0.498
Platelet, x10^6^, mean (SD)	185 (111)	233 (114)	< 0.001
BUN/Cr ratio, mean (SD)	32.3 (36.8)	31.5 (18.7)	0.591
Albumin, g/dL, mean (SD)	3.0 (0.6)	3.3 (0.7)	<0.001
INR, mean (SD)	1.9 (2.3)	1.5 (1.0)	<0.001

*BP* blood pressure, *BUN* blood urea nitrogen, *COX-2* cyclooxygenase-2, *Cr* creatinine, *INR* international normalized ratio, *NSAIDs* nonsteroidal anti-inflammatory drugs, *UGIB* upper gastrointestinal bleeding

For the total cohort, the mean age was 64.3 years, with a male predominance of 65%. The patients in the intervention group were younger, and male patients with coexisting cirrhosis and active malignancy were more prevalent. Furthermore, patients in the intervention group came to the hospital earlier. They required a higher rate of resuscitation, which was consistent with a significantly lower systolic blood pressure, a higher heart rate, more red emesis, a lower platelet number, a lower albumin level, and a higher INR level. For medication, the use of NSAIDs, COX-2 inhibitors, and clopidogrel was comparable between the two groups, but the nonintervention group consisted of higher aspirin and anticoagulant users.

### Model parameters

After analyzing the data for the model development, some independent parameters were correlated with each other. For example, patients with a history of stroke or heart disease tend to use antiplatelet agents; creatinine levels could reflect chronic kidney disease; syncope and altered mental status can be evaluated as unstable vital signs. These categorical parameters, such as the history of stroke, heart disease, chronic kidney disease, syncope, and alteration of consciousness, were dropped out because of their probable co-linearity by logical assumption, as they displayed equivalent properties of the subjects and caused unstable coefficient estimates or overfitting models. We performed the Variance Inflation Factor (VIF) to evaluate the co-linearity of the numerical parameters. The result showed that hemoglobin, systolic blood pressure, heart rate, and albumin had high VIF. However, they were crucial parameters used in many previous prediction models [31–37]. Therefore, these parameters were retained in the models. Several types of antithrombotic drugs were grouped as the preliminary models showed similar precision between the grouped parameters and the distinct parameters of this medication.

Finally, 18 parameters, including age, sex, presence of cirrhosis, active malignancy, use of antithrombotic drugs, previous history of UGIB, vomitus characters (red emesis or coffee-ground emesis), and stool characters (melena, maroon stool or red stool), duration of UGIB before hospitalization, resuscitation requirement, systolic blood pressure, heart rate, hemoglobin level, platelet number, serum albumin level, blood urea nitrogen level, creatinine level and INR level were used as input for the machine learning models. From 8 categorical data and 10 numerical data, all categorical features were transformed by the One-Hot Encoding method. Each categorical level becomes a separate feature in the dataset containing binary values, either 0 or 1. In doing so, the total of eighteen features was extended to twenty-one features. The blood urea nitrogen level and creatinine level will be computed as a ratio for machine learning analysis.

As mentioned above, 80% of the cohort (1,111 patients) was used as a training set for machine learning models. The baseline characteristics of the training set and the test set are shown in Table 2. There were no significant differences in patient profile, bleeding presentation, laboratory results, and endoscopic hemostatic intervention between these two sets.

**Table 2:** Clinical characteristics and intervention of the training set and the test set

	Training set (n = 1,111)	Test set (n = 278)	*P*-Value
**Baseline characteristics**			
Age, mean (SD)	64.2 (15.2)	64.8 (14.8)	0.559
Male, n (%)	720 (64.8)	182 (65.5)	0.892
Cirrhosis, n (%)	351 (31.6)	86 (30.9)	0.889
Malignancy, n (%)	176 (15.8)	55 (19.8)	0.137
Antithrombotic use, n (%)	556 (50.0)	133 (47.8)	0.555
Previous UGIB, n (%)	198 (17.8)	52 (18.7)	0.798
**Clinical presentation**			
Time to presentation (hour), mean (SD)	25.9 (23.6)	26.9 (22.6)	0.499
Red emesis, n (%)	333 (30.0)	77 (27.7)	0.503
Coffee ground, n (%)	366 (32.9)	86 (30.9)	0.570
Melena, n (%)	555 (50.0)	147 (52.9)	0.421
Maroon, n (%)	80 (7.2)	24 (8.6)	0.494
Red stool, n (%)	21 (1.9)	3 (1.1)	0.449
Systolic BP, mean (SD)	115.0 (25.7)	113.9 (22.9)	0.470
Heart rate, mean (SD)	92.2 (19.3)	93.7 (19.3)	0.247
Resuscitation, n (%)	245 (22.1)	57 (20.5)	0.632
**Laboratory findings**			
Hemoglobin, mean (SD)	8.2 (2.6)	8.1 (2.6)	0.935
Platelet (x10^6^), mean (SD)	210 (111)	218 (130)	0.375
INR, mean (SD)	1.7 (1.9)	1.7 (1.5)	0.680
Albumin, mean (SD)	3.1 (0.7)	3.2 (0.6)	0.127
BUN/Cr ratio, mean (SD)	32.0 (30.6)	31.4 (16.5)	0.648
**Result**			
Intervention, n (%)	482 (43.4)	133 (47.8)	0.204

*BP* blood pressure, *BUN* blood urea nitrogen, *COX-2* cyclooxygenase-2, *Cr* creatinine, *INR* international normalized ratio, *NSAIDs* nonsteroidal anti-inflammatory drugs, *UGIB* upper gastrointestinal bleeding

### Model performance

**Fig. 1:**
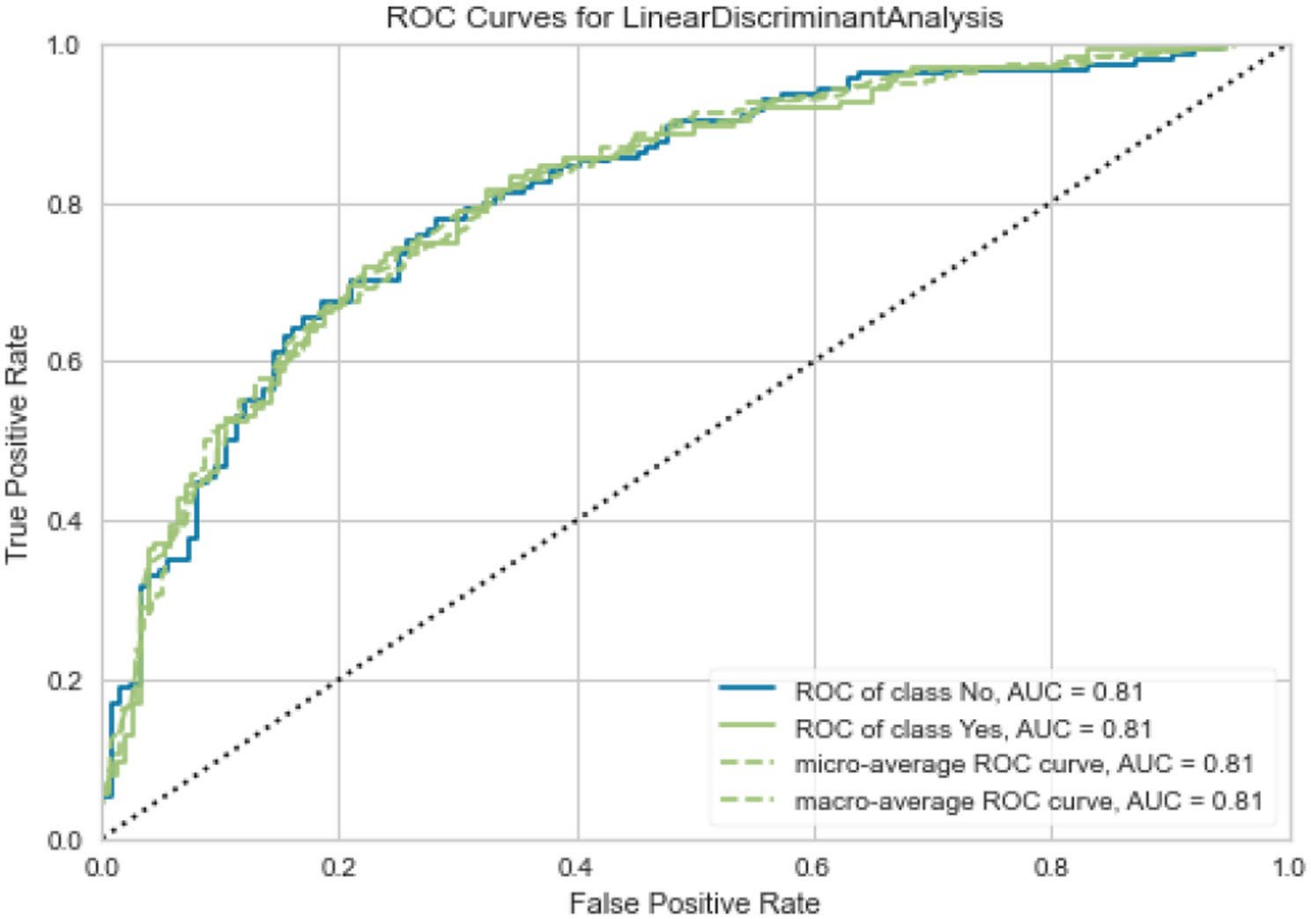
ROC curve for predicting endoscopic intervention based on Linear Discriminant Analysis model with the test set

According to the comparison results in Table 3, the linear discriminant analysis model demonstrated the highest AUROC, accuracy, and specificity. The AUROC of this model is 0.74, with a sensitivity of 57%, a specificity of 80%, and an accuracy of 70%. The result of the 5-fold cross-validation of the linear discriminant analysis model is shown in Supplementary Material Appendix 3. The model with the highest sensitivity of 63% was the Naïve Bayes model, but the AUROC was only 0.73. The linear discriminant analysis model was chosen for fine-tuning to develop the best performance, and its AUROC slightly increased to 0.75. For performance evaluation, the model was internally validated with the test set, which demonstrated an AUROC of 0.81, as shown in Fig. 1. The NPV and PPV of our model were calculated from the confusion matrix of the test set, and the results were 0.75 and 0.74, respectively, with a prevalence of UGIB of 0.44.

**Table 3:** Comparison of the average prediction performances of 15 models on the validation sets

Model	AUROC	Accuracy	Sensitivity	Specificity
Linear discriminant analysis	0.74	0.70	0.57	0.80
Logistic regression	0.74	0.70	0.58	0.79
CatBoost classifier	0.74	0.69	0.5	0.75
Gradient boosting classifier	0.73	0.67	0.58	0.75
Naïve bayes	0.73	0.69	0.63	0.75
Extra trees classifier	0.72	0.68	0.56	0.77
Random forest classifier	0.72	0.68	0.57	0.77
Decision tree classifier	0.72	0.67	0.59	0.78
Quadratic discriminant analysis	0.71	0.67	0.59	0.72
Ada boost classifier	0.71	0.67	0.60	0.73
Light gradient boosting machine	0.69	0.63	0.56	0.69
Extreme gradient boosting	0.69	0.63	0.56	0.68
K neighbors classifier	0.67	0.63	0.58	0.71
Lasso regression	0.62	0.63	0.49	0.75
Dummy classifier	0.50	0.56	0	1.0000

**Fig. 2:**
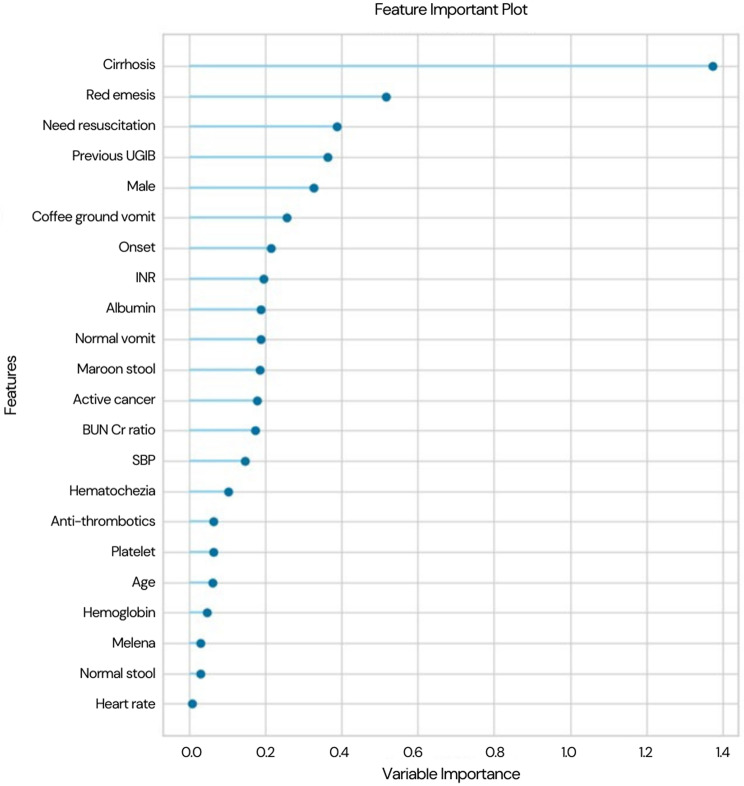
Feature importance plot for predicting endoscopic intervention based on the linear discriminant analysis model

**Fig. 3:**
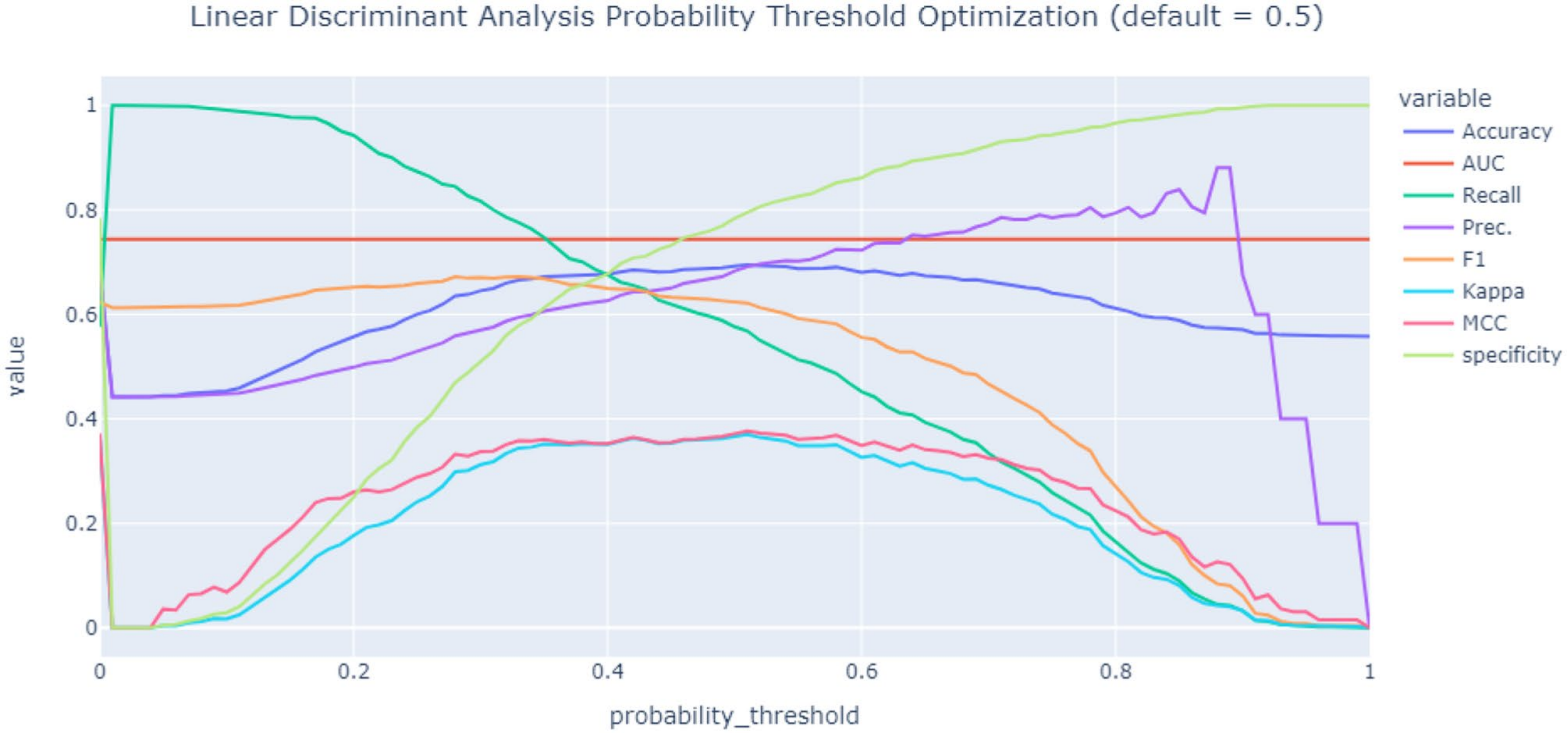
The threshold optimization plot of predicting endoscopic intervention based on Linear Discriminant Analysis model

The importance of the features was analyzed and shown in Fig. 2. Cirrhosis, red emesis, and the need for resuscitation were the three most important features of the model prediction of the need for endoscopic intervention. The probability threshold values to classify the patients into intervention groups or non-intervention groups are plotted in Fig. 3. The threshold of the model can be adjusted with the same AUROC result. At the default threshold of 0.5, our model has sensitivity and specificity of 74.5% and 81.4%, respectively. When the probability threshold value decreases, the model’s sensitivity increases, but the specificity decreases. For example, at the probability threshold of 0.17, the sensitivity could reach 99.2% with a specificity of 17.5%. After considering the accuracy in the figure, we found that the accuracy values were high when the probability threshold values ranged from 0.4 to 0.6, especially at values around 0.5. Therefore, this study used 0.5 as the threshold value to classify the patients into groups.

**Fig. 4:**
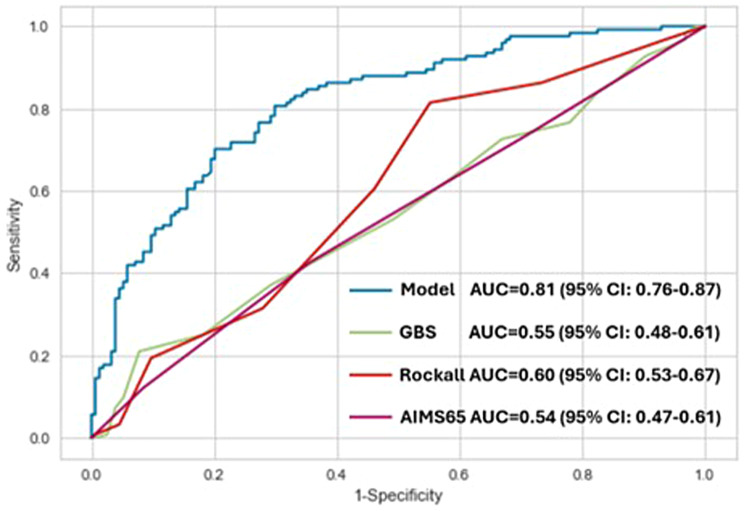
Comparison of the ROC curve for predicting endoscopic intervention among the developed model, Glasgow-Blatchford score, Rockall score, and AIMS-65 score

The developed Linear Discriminant Analysis model, GBS, pre-endoscopic RS, and AIMS65 score were applied to the test set cohort, and the AUROC of each score was analyzed, as shown in Fig. 4. The results showed that our model was superior to conventional scoring systems in predicting the need for endoscopic intervention (AUC, developed model 0.81 [95% CI 0.76–0.87] vs GBS 0.55 [95% CI 0.48–0.61] p < 0.001, pre-endoscopic RS 0.60 [95% CI 0.53–0.67] p < 0.001, AIMS65 score 0.54 [95% CI 0.47–0.61] p < 0.001).

**Fig. 5:**
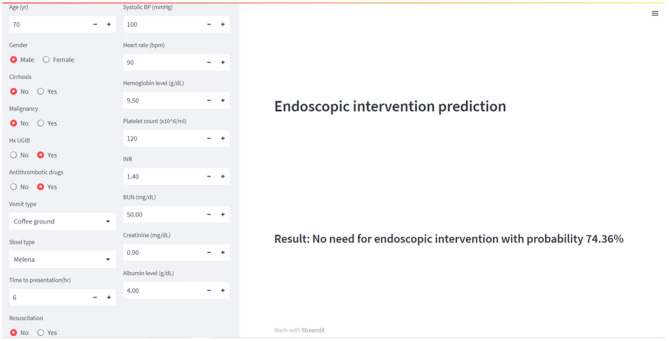
An application of endoscopic intervention prediction based on linear discriminant analysis (LDA) with an example of 18 features and their corresponding results

After we achieved the optimal prediction model based on linear discriminant analysis, we implemented this model in a local web application using Streamlit (Python Library), as demonstrated in Fig. 5. The panel on the left side will be used for data input. After inserting all the data, the result and probability of prediction will be instantly shown on the right side. This finalized program can be used on a local host computer.

## Discussion

Our study successfully developed a machine learning model to predict the need for endoscopic hemostatic intervention in patients with acute UGIB. The model was operated by entering 18 simple parameters, including 6 demographic data, 6 bleeding characteristic data, and 6 initial laboratory data. As confirmed by a large validation cohort, the accuracy of this model ranged from fair to good, with an AUROC of 0.81, an accuracy of 70%, a sensitivity of 57%, and a specificity of 80%. The results revealed that the linear discriminant analysis outperformed other machine-learning algorithms. This might be because our data is in a relationship in such a way that it could utilize the advantages of linear discriminant analysis. To illustrate this point, linear discriminant analysis (LDA) maximizes the separation between classes and helps reduce the overlap between classes in the reduced-dimensional space. This makes it highly effective for classification. Additionally, it helps prevent overfitting by reducing dimensionality while maintaining class-related information.

Prior studies have shown that the characteristics of active bleeding, such as red emesis and bloody gastric content, physical signs of hypovolemia, such as the presence of syncope, lower mean arterial pressure, and laboratory findings of low hemoglobin level, prolonged prothrombin time, and higher BUN level, were correlated with the performance of therapeutic intervention [31–33]. Combining those factors predicted endoscopic intervention with an AUROC comparable to all the parameters of GBS [33]. Subsequently, several scores were developed to exclusively predict endoscopic intervention in UGIB with parameters less complex than GBS (Table 4). The MAP(ASH) score was created in Spain and validated by a large cohort of the multiethnic population. The AUROC of the MAP(ASH) score was similar to the GBS but was significantly higher than the AIMS65 score [34]. Two Japanese scores were described: Nagoya University and H3B2 scores [35, 36]. Both of which demonstrated an AUROC higher than GBS. The London Haemostat Score (LHS) also showed higher accuracy than GBS [37]. Although these scoring systems provide similar or better accuracy to GBS with less complex parameters, their performance is still limited.

**Table 4:** Summary of studies that evaluated factors and scoring system in predicting endoscopic intervention and outcome in UGIB

Author (year)	Factors/model	Country	No. of factors	Derivative N (validation N)	Data collection	Intervention (%)	AUC Model	AUC GBS	Limitation
Lee CH [31]	Hematemesis, INR	Korea	2	270 (N/A)	2013–2017	59.0	N/A	N/A	OR 2.0–2.5
Kim SS [32]	Hb, bloody NG	Korea	2	613 (N/A)	2009–2013	53.7	N/A	N/A	OR 2.68, P < 0.001
Redondo CE [34]	MAP(ASH)	Spain	6	547 (3012)	2013–2017	40.8	0.61 (0.69)*	0.75	Fair performance
Veisman I [30]	Machine learning model	Israel	25	883 (N/A)	2012–2018	16.4	0.68	0.64	
Ito N [35]	Nagoya University Score	Japan	4	509 (160)	2016–2018	34.0	0.78	0.62	Exclude variceal bleeding
Sasaki Y [36]	H3B2 score	Japan	5	675 (N/A)	2015–2019	34.5	0.73	0.72	Exclude variceal bleeding
Marks I [37]	London Hemostat Score	UK	6	466 (404)	2015–2020	25.0	0.82 (0.8)*	0.72	Need calculation of positive and negative score
Acehan F [33]	Syncope, MAP, BUN	Türkiye	3	406 (N/A)	2019–2022	30.3	0.65	0.60	Exclude variceal bleeding

*AUC model of the validation set

An artificial model focusing on endoscopic intervention prediction has been developed to overcome the limited performance of scoring systems. Veisman et al. conducted a machine learning tool in Israel using 34 parameters to assess the possible correlation between baseline characteristics and endoscopic intervention in 883 patients [30]. The Random Forest model was created with a sensitivity, specificity, and AUROC of 0.55, 0.71, and 0.68, respectively. The AUROC of the model is higher than those of GBS (0.54) and pre-endoscopic RS (0.56), which is similar to the result of our population. The analysis plot showed that syncope, cirrhosis, and erythromycin use are correlated with the risk of intervention. Compared to our model, the sensitivity, specificity, and AUROC are higher than those of Veisman et al., with fewer parameters being used. The better performance of our model could be because we included a higher number of training populations for model derivation, and we tried creating a variety of models and selected the best performance among them. However, the significant parameters for the prediction were in the exact correlation. Cirrhosis is a common risk feature in both models, as it carries various mechanisms that potentiate endoscopic intervention, including esophagogastric varices, thrombocytopenia, and coagulopathy. UGIB patients with cirrhosis generally have varices and usually need endoscopic intervention for both therapeutic and prophylaxis purposes. The presence of red emesis or syncope and the requirement for fluid resuscitation may reflect the severity of bleeding from the underlying high-risk stigmata lesion that leads to endoscopic intervention. For erythromycin, the prokinetic effect was discussed to improve endoscopic visualization, and then the endoscopic intervention can be performed consecutively. However, the portion of erythromycin used in the study was small. In our center, intravenous erythromycin is unavailable, so our model did not include pre-endoscopic prokinetic use as a parameter.

In this study, the hemoglobin level was not an influential factor in predicting the endoscopic intervention. The explanation could be that it was only a one-time static parameter that did not represent the severity of bleeding. Many patients might have a baseline for chronic anemia from other medical conditions. From the previous model predicting endoscopic intervention by Veisman, et al. [30] and Ito et al. [35], hemoglobin was not correlated with increased risk for endoscopic intervention as well. The changes in hemoglobin level from baseline or the first test should be more accurately related to bleeding severity. However, this information was not available in our population.

Although numerous models were developed, the best model with great precision could not be achieved to predict the UGIB outcome. This could be explained by the hypothesis that UGIB is a complex disease with dynamic conditions, and the current risk scores are not dynamic [38]. The clinical severity could change from the first hospital visit to the endoscopy time due to the etiology of bleeding, patient comorbidity, anticoagulation, and resuscitation process. Pre-endoscopic medication, such as proton pump inhibitors, can downstage the endoscopic stigmata and reduce the need for endoscopic intervention [39]. A study comparing urgent endoscopy (<6 h) and early endoscopy (<24 h) in UGIB patients showed more high-risk stigmata lesions in the urgent group (66.4 vs. 47.8%). However, the mortality outcome was not different in both groups [40]. Therefore, static parameters at one time may not accurately predict the need for hemostatic intervention in UGIB. The intervention performed during index EGD may not represent the culprit lesion of that bleeding episode, and different physicians may also decide differently on the same lesion in each situation. For example, a cirrhotic patient with melena from a peptic ulcer, which at the time of endoscopy showed a clean base ulcer and a column of large esophageal varices with high-risk stigmata, may undergo rubber band ligation for primary prophylaxis as recommended in the clinical algorithm [41]. This could cause cirrhosis as the most crucial factor for model prediction. Furthermore, in real-life practice, patients who did not require hemostatic intervention still need endoscopic evaluation to diagnose the underlying etiology. Referral to an endoscopist is still mandatory but less urgent than a patient who needs hemostatic intervention.

**Fig. 6:**
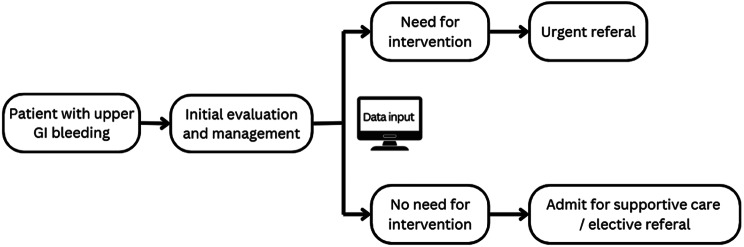
An algorithm for model implementation in primary care unit with the patients presented with upper GI bleeding

If the model were applied to the primary medical center, the patients with the initial diagnosis of UGIB would be evaluated and stabilized regularly. After inputting all parameters, the model will predict the endoscopic intervention probability of each patient. The physician would use this result to prioritize the urgency of an endoscopic intervention consultant or referral. Patients with low risk for intervention could be admitted for medical management and consulted for endoscopy later as elective or OPD cases. The proposed algorithm is shown in Fig. 6. For example, in our UGIB population, with a prevalence of 0.44 and NPV of 0.75, patients with predicted results as no need for intervention would have a 75% probability that they did not truly require urgent endoscopy. However, the physician should not miss the 25% risk. Other in-hospital evaluations, such as vital sign monitoring, serial hemoglobin level, and proper elevation of hemoglobin after blood transfusion, are still crucial for referral judgment. This protocol may reduce unnecessary urgent endoscopic procedures and optimize resource allocation in resource-limited settings.

The strength of our model is that we use numerous databases of more than 1000 patients with UGIB, including both variceal and nonvariceal bleeding. We generated 15 models and compared them to select the most productive model for prediction. Our model provided accuracy superior to that of the GBS. However, some limitations were noted in our model. First, this study included 10 years of data collection. Over the decades, the development and changes in practice may affect physicians’ decisions over time. However, the resuscitation and endoscopic treatment recommendations have not changed significantly since 2010 [42]. There are updates on restrictive blood transfusion strategies and new options for rescue therapeutic intervention, such as the over-the-scope clip and hemostatic spray, which do not affect the core concept of acute UGIB management. Second, about 30% of the patients with missing data were excluded, mainly due to missing the onset of symptoms and the INR. The characteristics of those missing data were compared to the eligible data and shown in Supplementary Material Appendix 4. Compared to the eligible population, the onset of symptoms and INR were not significantly different in both groups. There were significant differences in some parameters which might lead to bias. Also, some groups of patients were not included in the model computation, for example, the patients who did not undergo EGD due to low risk for GBS calculation, early death, or limitation of care. Moreover, some medical history not mentioned is considered to be absent such as unrecognized underlying disease, and untold over-the-counter NSAIDs or other medication. Third, certain critical factors that could affect the management decision were not included in the model, such as other anti-platelets in P2Y12 antagonists. They have been available in our center for the past few years. However, with high costs and reimbursement limitations, only a few patients received this medication. From our cohort, there were no patients prescribed these medications. The other crucial factors are time to endoscopy, pre-endoscopic medication, dynamic change in the bleeding characteristic or hemodynamic status after initial management, or the difference in hemoglobin level at present compared to baseline to assess the chronicity and severity of bleeding. In practice, these parameters are essential for the physician’s decision but require multiple data input steps and may not be practical in the primary setting. Further study, including multiple steps of data collection, would be helpful to maximize the model’s performance. Fourth, the CI95% for AUC, sensitivity, and specificity of the model were not presented as the results computed by Pycaret could not be further calculated to CI95%, but it was described in a similar pattern to the previous study [30]. Lastly, the management of UGIB patients relies on physician judgment. In our center, there were no hospital-specific protocols for physicians in the emergency department. Different physicians, including endoscopists, may act differently when encountering similar situations. The threshold for resuscitation can be varied in some cases. For example, the dynamic changes in clinical and laboratory parameters could lead to further resuscitation. Even though the management of endoscopists for intervention is based on the current guidelines mentioned in the method, some interventions depend on the endoscopist’s judgment, such as prophylaxis EV ligation in cirrhotic patients with non-variceal bleeding. In real-life applications, regardless of the accuracy of the models, physicians must combine these prediction results with other dynamic factors to get the most appropriate management for the patients.

## Conclusions

The prediction for endoscopic intervention in acute UGIB patients is complex and dynamic. In response to this challenge, our machine learning model, which used simple clinical parameters, performed fairly well in identifying UGIB patients who need endoscopic intervention. The practical implication of this study is that physicians in primary care units could prioritize patients who need a referral to endoscopic centers. Further development and external validation to identify more specific features will improve prediction performance.

## Electronic supplementary material

Below is the link to the electronic supplementary material.

Supplementary Material 1

## Data Availability

The datasets used and analyzed during the current study are available from the corresponding author upon reasonable request.

## Abbreviations

AUROC: Area under receiver operating characteristic
BP: Blood pressure
BUN: Blood urea nitrogen
COX-2: Cyclooxygenase-2
Cr: Creatinine
EGD: Esophagogastroduodenoscopy
GBS: Glasgow-Blatchford score
INR: International normalized ratio
NSAIDs: Non-steroidal anti-inflammatory drugs
RS: Rockall score
UGIB: Upper gastrointestinal bleeding
